# Single Incision non-thoracoscopic Nuss procedure for children with pectus excavatum: protocol for a multicenter, non-masked, randomized controlled trial

**DOI:** 10.3389/fsurg.2023.1210452

**Published:** 2023-07-19

**Authors:** Quan Wang, Zhengxia Pan, Chun Wu, Yonggang Li, Gang Wang, Jiangtao Dai, Chunnian Ren, Yiming Xie, Liangjun Xiong, Libing Zhang, Hongbo Li

**Affiliations:** ^1^ Department of Cardiothoracic Surgery, Ministry of Education Key Laboratory of Child Development and Disorders,National Clinical Research Center for Child Health and Disorders, China International Science and Technology Cooperation Base of Child Development and Critical Disorders, Children’s Hospital of Chongqing Medical University, Chongqing, China; ^2^ Chongqing Key Laboratory of Pediatrics, Chongqing Medical University, Chongqing, China; ^3^ Department of Pediatric Surgery, Chengdu Women and Children’s Central Hospital, Chengdu, China; ^4^ Department of Pediatric Surgery, Chongqing Three Gorges Central Hospital, Chongqing, China; ^5^ Department of Pediatric Surgery, Qujing Maternal and Child Health Hospital, Qujing, China

**Keywords:** pectus excavatum, single incision, Nuss procedure, multicenter, randomized controlled trial

## Abstract

**Background:**

Nuss procedure is the most common method of surgical treatment to pectus excavatum (PE). A significant percentage of surgeons choose to use thoracoscopic assistance during the Nuss procedure (TNP) to avoid cardiac injury. However, our previous findings confirm the safety of single incision Non-thoracoscopic Nuss Procedure (SINTNP). Hence, Further studies, particularly prospective randomized controlled trials, are necessary to assess the value of SINTNP for PE.

**Methods:**

This study is a prospective, superiority, multicenter, non-masked, randomized controlled trial that investigates the outcome and hospitalization medical expense of SINTNP compared to TNP for PE. A total of 320 eligible patients according to sample size calculation by retrospective data will be randomly assigned to the SINTNP group or the TNP group at a 1:1 ratio using stratified blocked randomization and the zone length was set as four. Patients aged between 3 and 18 years old for the first surgery and without combination of complex anomalies such as Marfan syndrome and congenital heart disease will be considered for the study. The co-primary endpoint is thoracic related complications and medical expense during hospitalization. Thoracic related complications were defined as pneumothorax, pleural effusion, pneumonia and incision infection. The secondary endpoints include surgery duration and length of hospital stay.

The registration number for this study protocol is ChiCTR230073081 (Chinese Clinical Trial Registry, A Primary Registry of International Clinical Trial Registry Platform, World Health Organization).

## 1. Background

Pectus excavatum is the most common anterior chest wall deformity in children (1). The Nuss procedure is the appropriate method for treating pectus excavatum in the majority of patients (2). Nuss initially reported a minimally invasive technique using bilateral incisions without the assistance of thoracoscopy (3). Subsequently, in a survey of members of the American Association of Pediatric Surgeons, 61% of members used thoracoscopic assistance during Nuss surgery to reduce the risk of cardiac or pulmonary injury (4). Dr. Nuss himself also emphasized that thoracoscopic assistance has been highly recommended since 1998 (5). Hence, the majority of surgeons tend to use thoracoscopic assistance in Nuss surgery (6), especially in adults (7).

However, the situation in the field of children may be different. Trained surgeons can safely pass bar through the space behind the sternum without relying on thoracoscopy in children because children's sternum is softer and more malleable. Only 49% of members of the Chinese Association of Thoracic Surgeons consider thoracoscopy to be a necessary method to avoid heart injury during the Nuss procedure (8). Additionally, if unilateral fixation can ensure the stability of the steel plate, then bilateral incisions are not necessary. Our center has been attempting modified single incision non-thoracoscopic Nuss procedure (SINTNP) since 2017 and has completed over 400 such surgeries to date, without any cardiac injury. The specific method of surgery can be found in our previous report (9, 10).

We speculate that modified single incision non-thoracoscopic Nuss procedure can reduce hospitalization costs, the proportion of thoracic-related complications, and the length of hospital stay in patients. Therefore, we conducted this multicenter randomized controlled trial to confirm the clinical value of SINTNP for pediatric patients compared with the thoracoscopic Nuss procedure (TNP) with bilateral incision.

## 2. Methods/design

**Figure 1:**
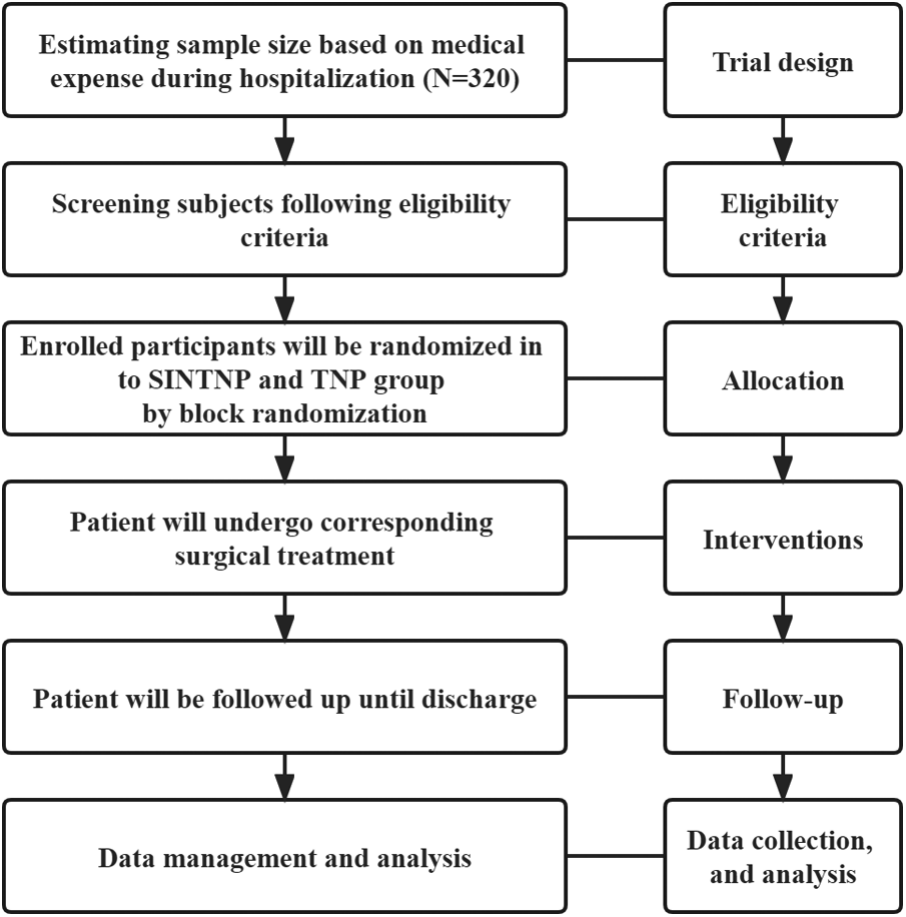
Flow chart of the study. SINTNP,single incision non thoracoscopic assisted Nuss surgery; TNP, thoracoscopic Nuss procedure.

This study aims to explore whether SINTNP can bring clinical benefits to patients with PE. All the design and analysis procedures will be carried out as shown in Figure 1, reported according to the Standard Protocol Items: Recommendations for Interventional Trials (SPIRIT) checklist (11) (Supplementary Table S1).

### 2.1. Objectives

#### 2.1.1. Primary objectives

The coprimary objectives of the study are to assess differences in thoracic-related complications and medical expenses during hospitalization of PE patients undergoing SINTNP or TNP.

#### 2.1.2. Secondary objectives

Secondary objectives were surgery duration and length of hospital stay.

### 2.2. Trial design and study setting

The study is a multicenter, non-masked, randomized controlled trial including patients with PE aged between 3 and 18 years old for the first surgery and without a combination of complex anomalies.

The participants will be randomly assigned to the SINTNP or TNP group with a balance of 1:1. Participants will be allocated as evenly as possible to four hospitals in western China. The sponsor institution was Children's Hospital of Chongqing Medical University. This multicenter study will also be conducted in Chengdu Women and Children's Central Hospital, Chongqing Three Gorges Central Hospital and Qujing Maternal and Child Health Hospital.

Due to objective conditions, the participants in the four centers cannot be completely balanced, but the participants in any center will not exceed 50% of the total number or be less than 15% of the total number. Independent statisticians will perform noninferiority tests for comparisons of SINTNP to TNP.

### 2.3. Eligibility criteria

#### 2.3.1. Inclusion criteria

The indications for surgical treatment included at least two of the following: (1) Haller Index (HI) greater than 3.20 or deformity had progressed by visual assessment of parents; (2) cardiac or pulmonary compression by visual assessment on CT or reduced cardiac diastolic function parameters such as E peak/A peak ratio and ventricular end diastolic volume index ratio on echocardiogram; (3) abnormal lung function by resting pulmonary function test; (4) mitral valve prolapse or arrhythmia; (5) symptoms such as repeated episodes of respiratory infections were present; (6) patients had a severe psychosocial disorder; and (7) patients or their parents had a strong desire for deformity correction.

#### 2.3.2. Exclusion criteria

Children who had not previously pass the developmental screening test such as Denver Developmental Screening Test (DDST) were not included. Patients with severe combined malformations, such as Marfan syndrome, severe cardiac malformations and Hirschsprung's disorder, were excluded. Patients who underwent secondary surgery were also excluded.

### 2.4. Interventions

After randomization into two groups, the enrolled patients will undergo routine preoperative preparation, including blood biochemical tests, and antibiotics will be administered once 30 min before surgery. All patients will undergo general anesthesia under tracheal intubation and receive T4 and T5 paravertebral nerve block. The SINTNP group will undergo left-side single-incision nonthoracoscopic-assisted Nuss surgery in accordance with our previous report (10). The TNP group will undergo thoracoscopic-assisted Nuss surgery with bilateral incisions, with the endoscopic observation hole on the right side. We need to emphasize that any child who requires the use of two bars during surgery will be withdrawn from the study cohort. After postoperative resuscitation, the patient will return to the ward and use a pain pump within 48 h after surgery.

One day after the surgery, chest radiograph bedside ward examination will be conducted. Starting from the second day after the surgery, a VAS pain score will be collected every morning. Those with a score of three or less will undergo the 100-meter walking experiment. Those who can successfully complete the 100-meter walking experiment independently and do not report any special discomfort will undergo chest radiograph examination. Those who had no obvious pneumothorax, pleural effusion, or steel plate displacement were discharged the next morning. If the child has a clear tendency toward plate allergy, such as extensive redness and swelling of the anterior chest wall skin or poor wound healing, such as incision suppuration, the patient will withdraw from the study. Their data will not be included in the analysis of pre-protocol set but will be truthfully reported and analyzed in the final research report of full analysis set.

### 2.5. Outcomes and participant timeline

The outcome indicators will be extracted from the CRF table after the patient is discharged and then uploaded to the online web-based database. Confirmation of outcome data will be undertaken with an additional sign-off by the site's principal investigator for each participant.

#### 2.5.1. Coprimary outcomes

Thoracic-related complications include pleural effusion, pneumothorax, and pneumonia diagnosed on chest radiograph.

Medical expenses during hospitalization are the total expenses recorded in the hospitalization electronic system, all of which are the original expenses before insurance reimbursement and are denominated in RMB.

#### 2.5.2. Secondary outcomes

The length of hospital stay is defined as a secondary outcome indicator, as it is a measure of the time cost for patients and their families.

The surgical duration affects the early recovery of children to a certain extent; therefore, it is defined as a secondary endpoint event, especially considering that this study was conducted using conventional tracheal intubation rather than tubeless techniques. Indeed, we emphasize that the long duration of the surgery cannot directly evaluate the quality of the surgical method.

#### 2.5.3. Participant timeline

The time schedule of enrollment, interventions, and assessments are described in detail in a schematic diagram (Table 1).

**Table 1:** Schedule of Enrolment, allocation, intervention and close-out.

Specific procedure	Enrolment	Allocation		Intervention					Close-out
Time point	D0	D0	D1	D2	D3	D4	Every day	DN	
Eligibility screen	●								
Informed consent	●								
Block randomization		●							
Preoperative examination			●						
Undergo surgery				●					
Analgesic pump				●	●				
Chest radiograph					●			●	
VAS score						●	●	●	
Walking experiment								●	
Data management									●

● The green dot indicates that this project will take place at this time point.

### 2.6. Sample size and recruitment

Medical expense during hospitalization is the primary outcome indicator of this study because it can objectively evaluate the financial burden of the patient's family. Based on our retrospective research data on surgeries over the past three years, the average cost of the TNP group was 30,250 RMB, while the average cost of the SINTNP group was 28,157 RMB. The standard deviation will be set as 2,000, and the superiority margin will be set as 1,500. The sample size will be calculated to require at least 141 children in each group.

The proportion of thoracic-related complications was approximately 9.6% in SINTNP and 13.6% in nonthoracoscopic-assisted Nuss surgery with double incision. The sample size calculation requires over 3,000 people per group according to the proportion of thoracic-related complications, and currently, we are unable to recruit so many patients. We will truthfully describe this recruitment-related limitation in the officially published article. Considering a 10% to 15% withdrawal and dropout rate, each group will ultimately include 160 children.

### 2.7. Sequence generation and random assignment

Block randomization will be performed, and the block length will be randomly set to any one of 4/6/8. The randomization of block length and intrablock randomization will be generated using EXCEL random numbers. The intrablock randomization values will be set to 1 and 2, representing two groups. The function formula is fx = RANDBETWEEN (1,2), and the block length randomization function formula is fx = 2 * RANDBETWEEN (2,4).

### 2.8. Allocation concealment and implementation

In each center, an authorized research nurse in the clinical unit will be responsible for recruiting the patient. All of the children's guardians signed a consent form. The envelope method will be used for allocation concealment, and the generated random allocation sequence will be placed in a sequentially encoded, sealed, and opaque envelope by Allocator 1. Allocator 1 will be unaware of the corresponding groups represented by randomized values 1 and 2 in advance. After determining the eligibility of the test subjects, Allocator 2 will open the envelopes in order and assign the test subjects to the corresponding experimental groups. After completing the allocation, the allocator will withdraw from this study and will not communicate with any participants or medical staff.

### 2.9. Masking

Radiologists, nurses, and data analysts will be blinded to group information. The label of group was open for doctors and family members of children's guardians. The outcome will be assessed blindly by a statistician who is blinded to the group information. After data analysis, the results of the group information will be unblinded by the director of the trial. At this point, all participants in the study can receive detailed labels of grouping information.

### 2.10. Data collection and management

A case report form (CRF) will be created in advance. All data will be entered into the CRF first, and then double data entry will be used to input data into the computer.

Each medical center will designate two investigators to gather patient baseline, outcome, and other relative trial data by electronic medical record. All electronic data and paper-based materials will be summarized. The characteristic table is shown in Table 2.

**Table 2:** Characteristic table of individual patients.

Characteristic	Age (years old)	Gender	Haller index	Pulmonary function	Cardiac function	Expenses during hospitalization (RMB/Yuan)	Thoracic-related complications	Surgery duration (minutes)	Length of hospital stay (day)
		Male		Normal	Normal		Pleural effusion		
		Female		Abnormal	Abnormal		Pneumothorax		
							Pneumonia		

Abnormal lung function is defined as (FEV1) less than 80% predicted value or FVC less than 80% predicted value or FEV1/FVC less than 92% predicted value.

Abnormal cardiac function is defined as mitral valve prolapse or arrhythmia.

All researchers underwent standardized training in advance to ensure that their medical services and data collection were standardized and that there were no significant differences between researchers. Only researchers who have passed the assessment of committee board can join this study. During the patient recruitment stage, all communication regarding the research background and process is strictly carried out in accordance with the section in the informed consent form. HL, LZ, YX and LX were members of the research committee board and are responsible for coordinating the entire research and handling difficulties such as inconsistent data. A multicenter meeting will be held every two weeks to ensure the smooth progress of the research.

### 2.11. Statistical methods

Continuous data will be presented as the means with standard deviations (SDs) or medians with interquartile ranges (IQRs). The Kolmogorov–Smirnov test will be used to evaluate normality. Continuous data will be compared between the two groups by unpaired t test (normal distribution) or the Mann‒Whitney *U*-test (nonnormal distribution). A chi-square test will be used to compare categorical variables between two groups. Pre-protocol analysis (PP) will be performed with data of participants finishing the whole study and intention to treat analysis (ITT) will be used in full analysis set. Inter group analysis across centers were performed to evaluate the influence of bias and the reliability of the overall study.

### 2.12. Monitoring

A Data Monitoring Committee (DMC) will be established to ensure the wellbeing of study participants. The DMC will periodically review study progress and outcomes as well as reports of unexpected serious adverse events (SAEs). The DMC will, if appropriate, make recommendations to the Trial Steering Committee (TSC) regarding continuance of the study or modification of the study protocol.

When any SAEs occur, the researcher will immediately report it to the committee and be authorized by the committee to terminate the study. SAEs include (1) incision infection; (2) bar exposure; (3) bar displacement; (4) VAS score higher than 8 after using analgesic; and (5) obvious bar allergy. All adverse events will be truthfully recorded in the CRF form and reported in the final research results.

Independent investigators from the Ethics Committee of Children's Hospital of Chongqing Medical University will review the standardization of the study every two weeks and randomly observe the process of patient recruitment, doctor‒patient communication, surgical implementation, and outcome evaluation by researchers. Investigators will be independent to researchers and sponsors and will not sign their names in the final manuscript. We will thank them for their contributions to the research in the acknowledgment section.

### 2.13. Ethics and dissemination

This study was approved by the Ethics Committee of Children's Hospital of Chongqing Medical University (approval number: 2022-325) and was registered in the Chinese Clinical Trail registry. The version number of this protocol is 2023-06, and the version is confirmed on April 8, 2023. The protocol may be slightly modified during the submission stage based on the comments of the reviewers and editors. After the publication of the research protocol, any form of modification of the research protocol is prohibited in this study. All of the children's guardians signed a consent form confirming participation in the study and authorizing the authors to collect the clinical data and publish potential research findings. This study will not conduct additional collection of biological samples from patients.

We will not record the name and contact information of the patient but instead use numerical numbers. Patient personal information will not be disclosed to any institution or individual. There were no financial or other competing interests for principal investigators for the overall trial and each study site.

The research committee board composed of HL, LZ, YX and LX has access to the final trial dataset. After the final research report is published, we will fully disclose the final trial dataset. Both surgical methods used in this study have been proven to be safe and reliable and are not expected to pose additional risks to patients. If the patient in this study suffers serious complications, we will provide all necessary assistance and compensation according to regulations.

Our final data will be attached to the final article for publication. Once the article is published, we will inform trial results to participants, health care professionals, the public, and other relevant groups via email.

## 3. Results

Since this is a protocol of multicenter prospective cohort study, the formal study has not yet started. We are unable to report the corresponding results. When the formal research is completed and published, we will refer to this protocol, and all results will be disclosed in the formal report.

## 4. Discussion

Heart injury is the most dangerous complication of pectus excavatum, so all surgeons will do their best to avoid it (12). A practical and feasible approach is to observe through thoracoscopy (13). Through thoracoscopy, the surgeon can clearly observe the process of tunnel establishment and bar passing through the sternum. Aberrant vessels detected in the mediastinal pleura could be evaded under direct visualization during mediastinal dissection with thoracoscopy (14). Additionally, it is traditionally believed that the bar needs to be fixed bilaterally to avoid displacement, so thoracoscopy usually does not add additional incisions. Thoracoscopic assistance is necessary in adults, especially in patients with recurrence, history of previous thoracic procedure or double-bar insertion (7, 15).

However, for children who undergo their first surgery and only need to use one bar in preoperative evaluation, thoracoscopic assistance may be redundant. Children's sternum is relatively soft and malleable, and separation behind the sternum is usually safe. Especially considering the recent use of single-incision Nuss surgery, thoracoscopic assistance is difficult and may not be necessary (10). We are worried that the thoracoscope entering the pleural cavity will cause additional pulmonary complications. Excessive pain and prolonged hospital stay time may reduce the quality of life of the patient and parents after surgery, while also increasing the workload and burden of clinical doctors. We hope to reduce the surgical costs and postoperative hospital stay of the patient. In a relatively underdeveloped region such as western China, a considerable proportion of patients still struggle to bear the cost of surgery (16). Therefore, we must strive to reduce their burden.

In the study, we only compare SINTNP to TNP and do not focus on the comparison between double incision non-thoracoscopic Nuss Procedure (DINTNP) to TNP. This is mainly due to two reasons. Firstly, because TNP requires two incisions, if we also use two incisions in non-thoracoscopy assisted Nuss surgery, then we do not utilize the advantages of non-thoracoscopy assisted surgery. Theoretically, DINTNP takes longer and has more injuries compared with single incision. The other reason is that our previous research has confirmed that the SINTNP will not increase the probability of postoperative bar displacement compared with the DINTNP. SINTNP is safe and reliable. Therefore, the current research setup is more in line with clinical scenarios.

Hence, we conducted this multicenter randomized controlled study. We believe that the results of this study are generalizable to other populations or healthcare settings. The learning curve of Nuss procedure is not long. Most thoracic surgeons can master this technique skillfully after a 10-procedure proctoring period (17). Non Thoracoscopy assisted single incision surgery has been carried out in many institutions. In addition, perioperative evaluation and nursing methods are also very common. The vast majority of institutions can perform preoperative CT, lung function, and echocardiography on children. Previous literature did not report significant cross regional or racial differences in the prognosis of Nuss surgery. Therefore, we believe that the results study can be widely generalized.

It is worth noting that although all researchers have undergone standardized training and each center's research will strictly follow the protocol. The potential bias in patient recruitment, protocol implementation, and data collection in multicenter studies cannot be completely avoided. We would like to confirm that for children with pectus excavatum who undergo their first surgery and need to use only one bar, the single-incision nonthoracoscopic Nuss procedure is sufficient. Other methods for ensuring safety, such as thoracoscopy or bilateral incision, are unnecessary.
